# The Novel Anti-Cancer Agent, SpiD3, Is Cytotoxic in CLL Cells Resistant to Ibrutinib or Venetoclax

**DOI:** 10.3390/hemato5030024

**Published:** 2024-08-27

**Authors:** Alexandria P. Eiken, Elizabeth Schmitz, Erin M. Drengler, Audrey L. Smith, Sydney A. Skupa, Kabhilan Mohan, Sandeep Rana, Sarbjit Singh, Jayapal Reddy Mallareddy, Grinu Mathew, Amarnath Natarajan, Dalia El-Gamal

**Affiliations:** 1 Eppley Institute for Research in Cancer and Allied Diseases, University of Nebraska Medical Center, Omaha, NE 68198, USA; 2 Fred and Pamela Buffett Cancer Center, University of Nebraska Medical Center, Omaha, NE 68198, USA

**Keywords:** chronic lymphocytic leukemia (CLL), drug resistance, SpiD3, ibrutinib, venetoclax, unfolded protein response (UPR), ferroptosis, oxidative stress

## Abstract

**Background:**

B-cell receptor (BCR) signaling is a central driver in chronic lymphocytic leukemia (CLL), along with the activation of pro-survival pathways (e.g., NF-κB) and aberrant anti-apoptotic mechanisms (e.g., BCL2) culminating to CLL cell survival and drug resistance. Front-line targeted therapies such as ibrutinib (BTK inhibitor) and venetoclax (BCL2 inhibitor) have radically improved CLL management. Yet, persisting CLL cells lead to relapse in ~20% of patients, signifying the unmet need of inhibitor-resistant refractory CLL. SpiD3 is a novel spirocyclic dimer of analog 19 that displays NF-κB inhibitory activity and preclinical anti-cancer properties. Recently, we have shown that SpiD3 inhibits CLL cell proliferation and induces cytotoxicity by promoting futile activation of the unfolded protein response (UPR) pathway and generation of reactive oxygen species (ROS), resulting in the inhibition of protein synthesis in CLL cells.

**Methods:**

We performed RNA-sequencing using CLL cells rendered resistant to ibrutinib and venetoclax to explore potential vulnerabilities in inhibitor-resistant and SpiD3-treated CLL cells.

**Results:**

The transcriptomic analysis of ibrutinib- or venetoclax-resistant CLL cell lines revealed ferroptosis, UPR signaling, and oxidative stress to be among the top pathways modulated by SpiD3 treatment. By examining SpiD3-induced protein aggregation, ROS production, and ferroptosis in inhibitor-resistant CLL cells, our findings demonstrate cytotoxicity following SpiD3 treatment in cell lines resistant to current front-line CLL therapeutics.

**Conclusions:**

Our results substantiate the development of SpiD3 as a novel therapeutic agent for relapsed/refractory CLL disease.

## 1. Introduction

Chronic lymphocytic leukemia (CLL) is a prevalent and incurable disease characterized by the accumulation of mature CD5^+^ B-cells in the lymph nodes, bone marrow, peripheral blood, and spleen [1]. CLL cells are highly dependent on aberrant apoptosis mechanisms (e.g., BCL2 upregulation) and pro-survival signaling, such as B-cell receptor (BCR) and NF-κB pathways for disease progression [1–6]. Lead front-line targeted therapies for CLL include small-molecule inhibitors that either target BCR signaling through the inhibition of Bruton’s tyrosine kinase (BTK), such as ibrutinib, or target apoptosis mechanisms such as the BCL2 antagonist, venetoclax [1]. Despite impressive initial clinical activity of these targeted therapies, persisting CLL cells lead to relapse in ~20% of patients, indicating a need for novel therapeutic development [7].

Clinically, most ibrutinib-resistant patients (~80%) harbor a C481S mutation in the BTK protein, blocking ibrutinib from covalently binding to BTK, and/or a gain of function mutation in PLCγ2, activating downstream BCR signaling independent of BTK inhibition [8]. Resistance is also mediated through alternative survival pathways, such as the activation of PI3K/AKT/ERK signaling [9,10]. Conversely, venetoclax resistance can be driven by the upregulation of other anti-apoptotic BCL2 family members such as BCL-xL and MCL1 by NF-κB activation [11]. However, directly targeting BCL-xL or MCL1 leads to significant adverse effects in patients, such as thrombocytopenia and hepatotoxicity, respectively [12,13]. Additionally, reprogramming of the mitochondrial outer membrane through prevention of BAX/BAK oligomerization can result in altered expression of BCL2 family members and increased oxidative phosphorylation (OXPHOS) activity, both of which lead to venetoclax resistance [14]. Lastly, interactions between CLL cells and their microenvironment likely contribute to intrinsic drug resistance, in part through the upregulation of NF-κB signaling and anti-apoptotic genes [15–17].

Ferroptosis is a distinct regulated cell death mechanism induced by the iron-dependent peroxidation of polyunsaturated fatty acids (PUFAs) [18–20]. The system cysteine/glutamate antiporter Xc−, consisting of the antiporters SLC7A11 and SLC3A2 and their axis with glutathione (GSH), and glutathione peroxidase 4 (GPX4), is the main pathway that prevents the onset of ferroptosis through the import of cyst(e)ine. Cyst(e)ine is used to synthesize GSH, which GPX4 uses to detoxify lipid peroxides [18–20]. The deprivation of cyst(e)ine and consequent GSH depletion leads to the accumulation of peroxidized lipid species, culminating to cell death [21,22]. Recently, studies have shown that inducing ferroptosis could activate cell death in leukemia and lymphoma cells. For example, the p53-activator, APR-246, can trigger ferroptosis-induced cell death in AML and TP53-mutated diffuse large B-cell lymphoma (DLBCL) [23,24]. Dimethyl fumarate can induce lipid peroxidation, which triggers ferroptosis in germinal center DLBCL [25]. Lastly, ferumoxytol, an iron oxide nanoparticle, can induce lipid peroxidation through the accumulation of reactive oxygen species (ROS) in DLBCL cells [26]. Studies on the role of ferroptosis and its impact on pathophysiology and therapeutic development in CLL have been sparse. Thus far, ferroptosis-related prognostic scores have been identified in CLL patients [27–29], and a handful of studies have demonstrated the sensitivity of CLL cells to ferroptotic cell death [30,31]. Since CLL cells have elevated amounts of ROS compared to healthy lymphocytes [32], they may be more vulnerable to agents that further increase ROS and oxidative stress. Similarly, excessive concentrations of ROS can result in a buildup of polyubiquitylated proteins [33], leading to the induction of the unfolded protein response (UPR) in addition to ferroptosis.

Analog 19 is a spirocyclic compound that displays NF-κB inhibitory activity with antitumor effects [34–36]. Spirocyclic dimers of analog 19 (SpiDs) resulted in improved NF-κB inhibitory activity by crosslinking key members of the NF-κB complexes [37–39]. Recently, we showed that SpiD3 exhibited potent preclinical anti-leukemic effects, exerting multifactorial CLL cytotoxicity through diminished NF-κB signaling and protein translation [40]. Our prior studies show SpiD3 generated an immense unfolded protein load by covalently binding to surface-exposed cysteine (SEC) residues, inducing the UPR and subsequently inhibiting protein translation. Relatedly, SpiD3 treatment also led to the accumulation of cytotoxic ROS, which was prevented with antioxidant pre-treatment, partially restoring CLL cell viability. Lastly, we have shown that SpiD3 synergizes with ibrutinib and is effective in inhibiting ibrutinib-resistant CLL cell survival and proliferation. Prompted by these impressive preclinical results, we sought to uncover potential vulnerabilities of drug-resistant CLL cells by further investigating SpiD3’s mechanism of action in ibrutinib- and venetoclax-resistant CLL cell lines. We performed RNA-sequencing on inhibitor-resistant CLL cells and the matched parental/wild-type controls in the presence and absence of SpiD3, venetoclax, or ibrutinib. Cellular response to oxidative stress, autophagy, ferroptosis, MAPK signaling, and NRF2 pathways were among the top pathways modulated by SpiD3. In both ibrutinib- and venetoclax-resistant CLL cell lines, there was an increase in Ras/Raf/MAPK/ERK, WNT/β-catenin, and NOTCH signaling, highlighting a potential vulnerability in inhibitor-resistant CLL. Functionally, SpiD3 markedly upregulated protein aggregation, ROS production, and lipid peroxidation in both inhibitor-resistant CLL cell lines, highlighting SpiD3’s unique mechanism of action and its clinical relevance as a potential therapeutic for drug-resistant CLL.

## 2. Materials and Methods

### 2.1. Pharmacological Agents, Inhibitors, and Stimulants

SpiD3 was synthesized at the University of Nebraska Medical Center (UNMC; Omaha, NE, USA) following reported procedures [39]. Thapsigargin, ibrutinib, venetoclax, and ferrostatin-1 were purchased from Cayman Chemical (Ann Arbor, MI, USA) and dissolved in DMSO. Iron (II) chloride tetrahydrate (FeCl_2_), purchased from ThermoScientific Chemicals (Waltham, MA, USA), was reconstituted in a solution of 0.1% bovine serum albumin and sterile water.

### 2.2. Cell Lines

The HG-3 CLL cell line was ordered from DSMZ (Braunschweig, Germany), and the OSU-CLL cell line [41] was provided by the Human Genetics Sample Bank of Ohio State University (Columbus, OH, USA). The molecular profile of the CLL cell lines is summarized in Supplementary Table S1. Ibrutinib-resistant (IR) HG-3 cells were generated by prolonged exposure (~3 months) of parental/wild-type (WT) HG-3 cells to increasing concentrations of ibrutinib, as previously described [42]. IR-HG3 cells sustained growth at 30 μM ibrutinib (maximum concentration). Venetoclax-resistant (VR) OSU-CLL cells were generated by prolonged exposure (~6 months) of WT-OSUCLL cells to increasing concentrations of venetoclax. VR-OSUCLL cells sustained growth at 20 μM venetoclax (maximum concentration). Before experimental use, inhibitor-resistant cells were incubated with their maximum inhibitor concentration for 24 h; thereafter, the inhibitors were washed out and the cells were left to recover (~2 days) [43]. All CLL cell lines were cryopreserved in large stock, used within 8 weeks after thawing, and cultured in RPMI-1640 with 2 mM L-glutamine (Sigma-Aldrich; St. Louis, MO, USA) supplemented with 100 U/mL penicillin/100 μg/mL streptomycin (P/S, Sigma-Aldrich; St. Louis, MO, USA) and 10% heat-inactivated fetal bovine serum (Avantor^®^; Radnor, PA, USA). Before experimental use, cell line cultures were confirmed to be free of mycoplasma using the MycoAlert kit from Lonza (Basel, Switzerland).

### 2.3. Cytotoxicity Assays

WT- or VR-OSUCLL cell lines (75,000 cells/well) were treated with vehicle (DMSO) or increasing inhibitor concentrations (single or combined) in a 96-well plate for 72 h. As an indicator of cell proliferation, mitochondrial activity was evaluated using the CellTiter 96^®^ Aqueous MTS assay (Promega; Madison, WI, USA), as previously described [40,42]. The absorbance signal from each well was measured at 490 nm using a Tecan Infinite^®^ M1000 Pro microplate reader (Männedorf, Switzerland). GraphPad Prism (v9.4.1; GraphPad Software, Inc.; San Diego, CA, USA) was used to calculate the half-maximal inhibitory concentration (IC_50_). To assess synergy, combination indices (CIs) were calculated using CompuSyn (v1.0) [44].

To measure cell viability and apoptosis, WT- or VR-OSUCLL cell lines (1 × 10^6^ cells/well) were treated in a 48-well plate with vehicle (DMSO) or increasing inhibitor concentrations for 24 h and measured using an Annexin V-FITC/propidium iodide (PI) assay kit (Leinco Technologies; Fenton, MO, USA) per the manufacturer’s protocol. Live cells were gated as Annexin V^−^/PI^−^. Annexin V^+^ cells were counted as the apoptotic fraction.

### 2.4. Immunoblot Assays

WT- and IR-HG3 cell lines were treated with SpiD3 (1, 2 μM), ibrutinib (1 μM), or FeC1_2_ (160 μM) for 4 h before protein extraction. WT- and VR-OSUCLL cell lines were treated with SpiD3 (0.5, 1, 2 μM), venetoclax (0.1, 1 μM), or FeC1_2_ (160 μM) for 24 h before protein extraction. Total cell protein was extracted using protein lysis buffer (20 mM Tris pH 7.4, 150 mM NaCl, 1% Igepal CA-630, 5 mM EDTA) containing protease and phosphatase inhibitor cocktails and phenylmethyl sulfonyl fluoride (Sigma-Aldrich; St. Louis, MO, USA). BCA protein analysis (ThermoFisher Scientific; Waltham, MA, USA) was used to determine equal concentrations of protein in whole-cell lysates. Samples were then denatured at 95 °C for 5 min in loading dye containing sodium dodecyl sulfate (SDS), separated by 1.5 mm SDS-PAGE gels, and then transferred onto nitrocellulose membranes using the Trans-Blot Turbo Transfer System (Bio-Rad; Hercules, CA, USA). Membranes were incubated overnight in primary antibody, washed, and incubated with HRP-conjugated anti-rabbit Ig or anti-mouse Ig (Cell Signaling Technology; Danvers, MA, USA) for 1 h. Membranes were then visualized on the ChemiDoc Imaging System (Bio-Rad; Hercules, CA, USA) following development with WesternBright^™^ ECL or Sirius (Advansta; San Jose, CA, USA) according to the manufacturer’s instructions. The primary antibodies used are listed in Supplementary Table S2.

### 2.5. RNA-Sequencing and Data Analysis

RNA was extracted from CLL cells (1.5 × 10^6^ cells/mL) treated with either DMSO, SpiD3 (2 μM), ibrutinib (1 μM), or venetoclax (1 μM) for 4 h using the miRNeasy Mini Kit (Qiagen; Hilden, Germany) per the manufacturer’s instructions and processed using the Universal Plus mRNA-Seq with NuQuant kit (Tecan; Männedorf, Switzerland). RNA quality was assessed on the Fragment Analyzer Automated CE System (Advanced Analytical Technologies, Inc; Ames, IA, USA), and RNA (250 ng) was pair-end sequenced (2 × 100 bp, 200 cycles) on Illumina NovaSeq6000 (San Diego, CA, USA) at the UNMC Genomics Core. Sequenced reads were mapped using STAR (v2.7.8) to genome-build hg38, guided by ENSEMBL GRCh38.99 transcript annotations. Transcript read counts were obtained using salmon (v1.9) [45]. Differential gene expression and normalized counts (variance stabilizing transformation) were obtained using DESeq2 [46], and volcano analysis was graphed using GraphPad Prism (v9.4.1). Genes with *FDR* < 0.05 and |Log_2_ FC| > 1 were considered significant and were analyzed using Metascape (https://metascape.org, accessed on 27 October 2023) [47] for gene set enrichment analysis (GSEA). Heatmaps were generated using Heatmapper.ca [48]. RNA-sequencing data are deposited at GSE267095. The transcriptional profile of vehicle- and 2 μM SpiD3-treated WT-OSUCLL samples from our previous study (GSE236239) [40] was analyzed with the vehicle- and SpiD3-treated WT-OSUCLL cells for the differential gene expression analysis and subsequent GSEA and volcano analysis.

### 2.6. Reactive Oxygen Species (ROS) Detection

Following a 1 h pre-treatment with 5 mM N-acetylcysteine (Cayman Chemical; Ann Arbor, MI, USA), CLL cell lines (1 × 10^6^ cells/mL) were incubated for 24 h with SpiD3 (0.5, 1, 2 μM), ibrutinib (1 μM), or venetoclax (1 μM) and then stained using the ROS-detection cell-based assay kit (DCFDA; Cayman Chemical; Ann Arbor, MI, USA) per the manufacturer’s protocol. Helix NP^™^ NIR (BioLegend; San Diego, CA, USA) was used for live/dead discrimination before flow cytometry analysis. The fold-change in median fluorescence intensity (MFI) was calculated as the treatment group MFI/control MFI.

### 2.7. Protein Aggregation

CLL cell lines (1 × 10^6^ cells/mL) were treated for 24 h with SpiD3 (0.5, 1, 2 μM), ibrutinib (1 μM), venetoclax (1 μM), or thapsigargin (2 μM) and then stained with 400 nM CRANAD2 (Cayman Chemical; Ann Arbor, MI, USA) for 30 min at 37 °C to assess cellular levels of protein aggregates. Ghost Dye^™^ Violet 450 (Cell Signaling Technologies; Danvers, MA, USA) was added for live/dead discrimination before flow cytometry analysis. Fold-change in MFI was calculated as the treatment group MFI/control MFI.

### 2.8. Ferroptosis Detection

CLL cell lines (1 × 10^6^ cells/mL) were pre-treated with the ferroptosis sparing agent ferrostatin-1 (10 μM) for 1 h [19] and then treated with either vehicle (DMSO), SpiD3 (0.5, 1, 2 μM), ibrutinib (1 μM), venetoclax (1 μM), or FeCl_2_ (160 μM) for up to 48 h. Ferroptotic cell death was monitored by flow cytometry using Zombie NIR (BioLegend; San Diego, CA, USA) for live/dead discrimination in combination with BODPIY-C11 (BC11) 581/591 probe (Invitrogen; Waltham, MA, USA) for the detection of lipid peroxidation. Changes in lipid peroxidation from viable cells were monitored by the percentage of PE^+^/FITC^+^ cells.

### 2.9. Flow Cytometry

Flow cytometry was performed on an LSRII (BD Biosciences; Franklin Lakes, NJ, USA) or NovoCyte 2060R (Agilent; Santa Clara, CA, USA) cytometer and analyzed using NovoExpress v1.3.0 (Agilent; Santa Clara, CA, USA) or Kaluza v2.1 (BD Biosciences; Franklin Lakes, NJ, USA).

### 2.10. Statistics

Data are reported as mean ± standard error of the mean (SEM). GraphPad Prism (v9.4.1) was used to perform unpaired *t*-tests with Welch’s correction for comparisons of two groups and one-way ANOVAs with Dunnett’s multiple comparison test for comparing more than two groups. *p* < 0.05 was considered significant.

## 3. Results

### 3.1. SpiD3 Induces a Unique Transcriptional Program in Ibrutinib-Resistant CLL Cells

**Figure 1.**
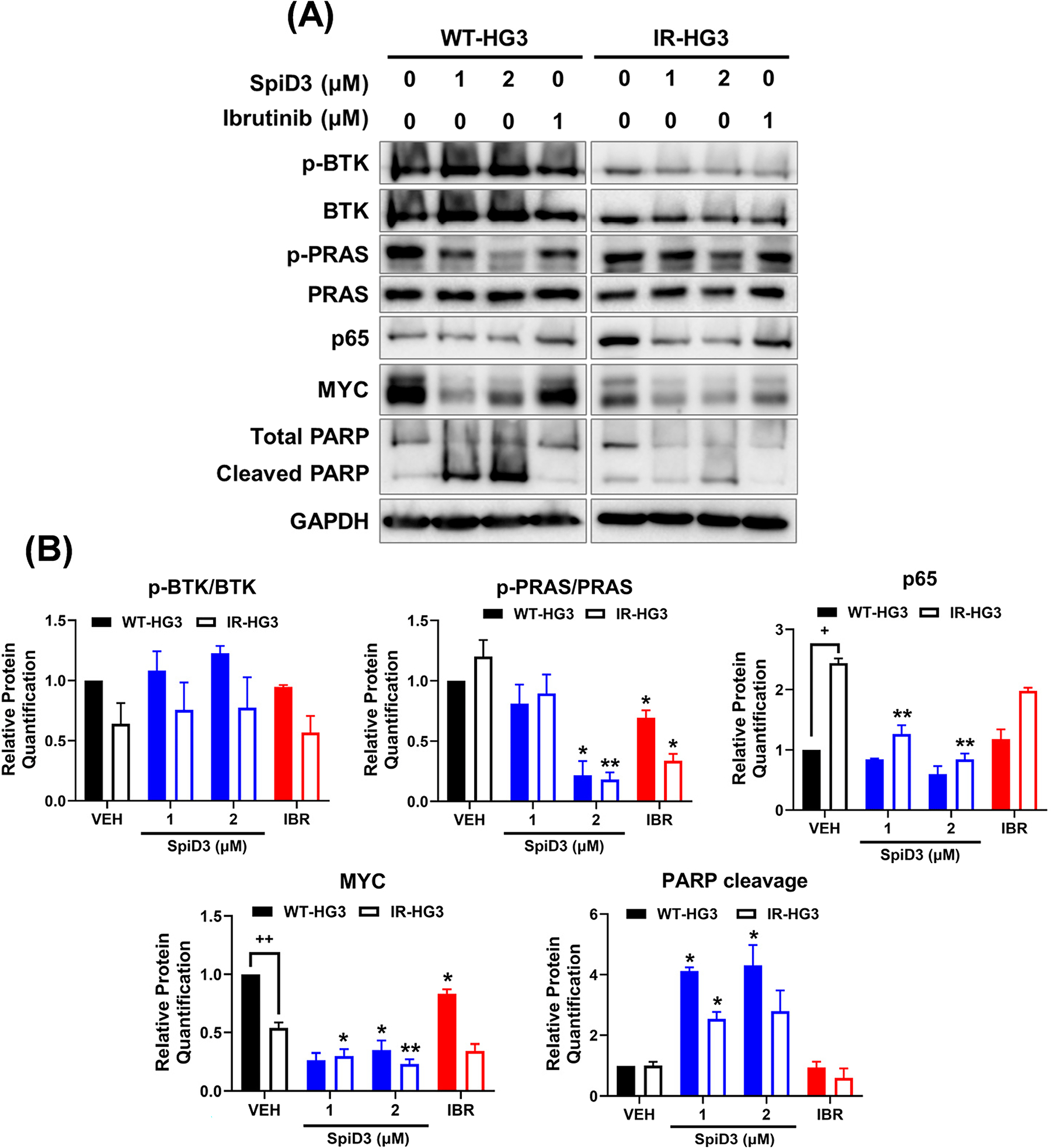
SpiD3 elicits cytotoxic effects in ibrutinib-resistant CLL cells. (**A**) Representative immunoblot analysis of p-BTK (Tyr223), total BTK, p-PRAS (Thr246), total PRAS, p65, MYC, and PARP (total and cleaved) from parental wild-type (WT) and ibrutinib-resistant (IR) HG-3 cells treated with SpiD3 (1, 2 μM), ibrutinib (IBR; 1 μM), or equivalent DMSO vehicle for 4 h (*n* = 3 independent experiments/cell line). GAPDH served as the loading control. (**B**) Protein quantification of the immunoblot analysis of p-BTK (Tyr223), p-PRAS (Thr246), p65, MYC, and cleaved PARP. Data are represented as mean ± SEM. Asterisks denote significance vs. corresponding VEH: * *p* < 0.05, ** *p* < 0.01. Plus signs denote significance between WT-HG3 and IR-HG3 cells: ^+^ *p* < 0.05, ^++^ *p* < 0.01.

We previously reported that SpiD3 synergizes with ibrutinib and retains its cytotoxic effects in the unmutated immunoglobulin heavy-chain variable region gene (*IGHV*) HG-3 CLL cells resistant to ibrutinib [40]. Herein, we show that SpiD3 induced PARP cleavage and decreased PRAS phosphorylation, MYC expression, and p65 expression in both WT- and IR-HG3 cells (Figure 1A,B). In contrast, ibrutinib did not decrease the protein expression of p65 or the phosphorylation of BTK or PRAS in IR-HG3 cells compared to WT-HG3 cells (Figure 1A,B).

**Figure 2.**
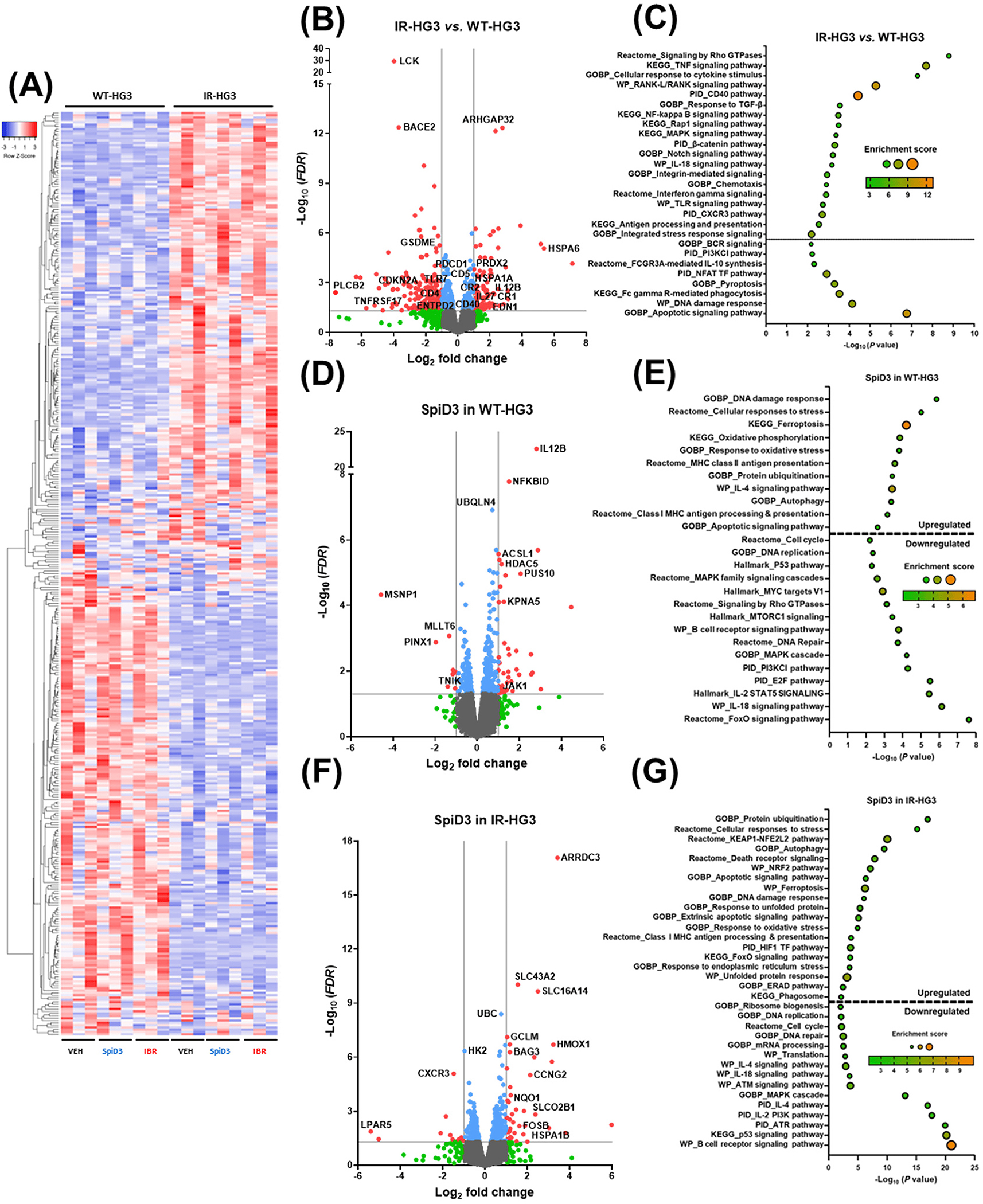
SpiD3 modifies transcriptional profiles in ibrutinib-resistant CLL cells. (**A**–**G**) RNA-sequencing of parental wild-type (WT) HG-3 and ibrutinib-resistant (IR) HG-3 cells treated with SpiD3 (2 μM), ibrutinib (IBR; 1 μM), or equivalent DMSO vehicle (VEH; *n* = 3 independent experiments). (**A**) Hierarchical clustering of the top 500 differentially expressed genes (DEGs) in VEH-treated IR-HG3 cells compared to VEH-treated WT-HG3 (*FDR* < 0.05). Red indicates increased gene expression (0 < z-score < 2), and blue indicates decreased expression (−2 < z-score < 0). (**B**,**D**,**F**) Volcano plots of VEH-treated IR-HG3 (**B**), SpiD3-treated WT-HG3 (**D**), or SpiD3-treated IR-HG3 cells (**F**) compared to VEH-treated HG-3 cells with select CLL-relevant genes labeled. Genes meeting both statistical significance (*FDR* < 0.05) and fold-change (|Log_2_ FC| > 1) parameters (red) were used for downstream analysis. Genes meeting only statistical significance (blue), only fold-change (green), or neither threshold (gray) are shown for comparison. (**C**,**E**,**G**) Gene set enrichment analysis of statistically significant DEGs in VEH-treated IR-HG3 (**C**), SpiD3-treated WT-HG3 (**E**), and SpiD3-treated IR-HG3 cells (**G**) compared to VEH-treated HG-3 cells.

RNA-sequencing analysis was conducted to further elucidate the mechanism of action of SpiD3 in ibrutinib-resistant CLL (Figure 2; Supplementary Figure S1). Transcriptional analysis revealed that over 1200 genes (*FDR* < 0.05) were modulated in untreated IR-HG3 cells compared to untreated WT-HG3 cells (Figure 2A,B). Various immune response-related genes, such as *CR1*, *IL-27*, *IL-12B*, and *CD40*, were increased in IR-HG3 cells (Figure 2A,B). Correspondingly, pathway enrichment analysis using Metascape [47] showed that “TNF signaling”, “cellular response to cytokine stimulus”, “T-cell activation”, “response to TGF-β”, “IL-18 signaling pathway”, and “interferon gamma signaling” were upregulated in IR-HG3 cells (Figure 2C). Many of these pathways lead to NF-κB activation, highlighting a possible therapeutic vulnerability in these ibrutinib-resistant cells. Intriguingly, genes involved in the “protein folding” and “integrated stress response” pathways such as *RAP1A*, *HSPA6*, and *HSPA1A* were upregulated in IR-HG3 cells when compared to their WT counterparts, indicating a potential cellular adaptive mechanism to mitigate increased cellular stress (Figure 2B,C). Notably, “BCR signaling” and “PI3K signaling” pathways were decreased in IR-HG3 cells, but pathways critical for CLL proliferation and survival, such as “signaling by Rho GTPases”, “MAPK signaling”, “WNT/β-catenin signaling”, and “NOTCH signaling”, were increased in the ibrutinib-resistant cells (Figure 2C). This suggests that the resistant cells rely on alternative signaling pathways for survival and to circumvent the ibrutinib treatment [49–51]. There was also a decrease in apoptosis-related genes (Figure 2B,C), indicating that IR-HG3 cells displayed more anti-apoptotic signaling.

Next, we evaluated the effects of ibrutinib and SpiD3 in IR-HG3 cells. Unsurprisingly, there were fewer genes modulated by ibrutinib treatment in IR-HG3 cells compared to WT-HG3 cells (95 genes vs. 246 genes, respectively, *FDR* < 0.05; Supplementary Figure S1A,C). “NF-kappa B signaling” and “BCR pathway” pathways were downregulated by ibrutinib in WT-HG3 cells, but not in IR-HG3 cells, illustrating the expected loss of ibrutinib’s effect on the resistant cells (Supplementary Figure S1B,D). Additionally, ibrutinib upregulated “membrane trafficking” and “DNA metabolic process” pathways in WT-HG3 cells but not in IR-HG3 cells (Supplementary Figure S1B,D). In both WT- and IR-HG3 cells, SpiD3 treatment increased genes involved in “cellular response to stress”, “autophagy”, “class I MHC antigen processing and presentation”, and “apoptosis” pathways (Figure 2D–G). SpiD3 also decreased genes related to “cell cycle”, “BCR signaling”, and “DNA repair” pathways in both cell lines (Figure 2D–G). Additionally, genes that regulate ferroptosis and the NRF2/KEAP1 pathway, such as *HMOX1*, *GCLM*, and *NQO1*, were upregulated with SpiD3 treatment in both the WT- and IR-HG3 cells (Figure 2D–G), indicating that SpiD3 could be involved in ROS production and induction of ferroptotic signaling. Excitingly, pathways that were upregulated in IR-HG3 cells compared to WT-HG3 cells, such as “MAPK signaling” and “signaling by Rho GTPase”, were downregulated with SpiD3 treatment in both cell lines (Figure 2D–G), suggesting that SpiD3 can target these heightened alternative survival mechanisms/pathways. These results highlight the ability of SpiD3 to modulate various pro-survival and anti-apoptotic pathways in ibrutinib-resistant CLL cells.

### 3.2. SpiD3 Modulates Critical Cancer Pathways in Ibrutinib-Resistant CLL Cells

**Figure 3.**
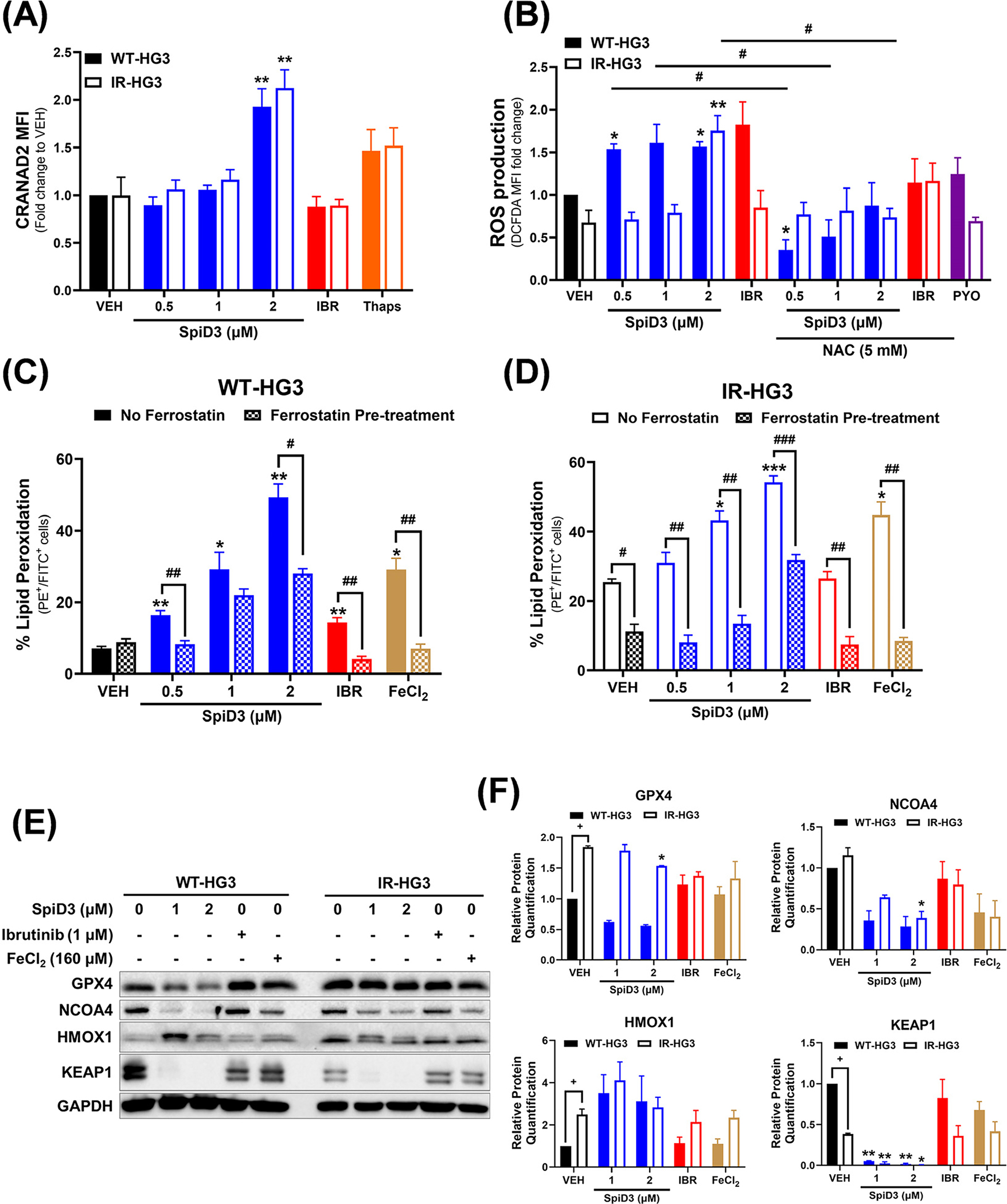
SpiD3 subverts oncogenic pathways in ibrutinib-resistant CLL cells. (**A**) Parental wild-type (WT) and ibrutinib-resistant (IR) HG-3 cells were treated for 24 h with SpiD3 (0.5, 1, 2 μM), thapsigargin (Thaps; 2 μM), ibrutinib (IBR; 1 μM), or equivalent DMSO vehicle (VEH) and then incubated with CRANAD2 dye to probe for aggregate proteins. Data are represented as mean ± SEM of the fold-change in CRANAD2 median fluorescent intensity (MFI) compared to WT-HG3 VEH (*n* = 3 independent experiments/cell line). Asterisks denote significance vs. VEH: ** *p* < 0.01. (**B**) WT-HG3 and IR-HG3 cells were pre-treated with 5 mM N-acetylcysteine (NAC, 1 h), followed by SpiD3 (0.5, 1, 2 μM), IBR (1 μM), or VEH (24 h). Pyocyanin (PYO, 1 mM) served as a control ROS inducer (n = 3 independent experiments/cell line). ROS production is represented as the fold-change in DCFDA MFI to VEH-treated WT-HG3 cells. Data are represented as mean ± SEM. Asterisks denote significance vs. corresponding VEH: * *p* < 0.05, ** *p* < 0.01. Hashtags denote significance between non-NAC pre-treated samples vs. NAC pre-treated samples: ^#^ *p* < 0.05. (**C**,**D**): WT-HG3 (**C**) and IR-HG3 (**D**) cells were pre-treated with 10 μM ferrostatin (1 h; inhibitor of lipid peroxidation) followed by SpiD3 (0.5, 1, 2 μM), IBR (1 μM), or VEH (48 h). Iron (II) chloride tetrahydrate (FeCl_2_; 160 μM) served as a control lipid peroxidation inducer (*n* = 3 independent experiments/cell line). Cells were analyzed by flow cytometry for changes in peroxidized lipid generation, observed by a change in fluorescence from PE to FITC. Data are represented as mean ± SEM. Asterisks denote significance vs. corresponding VEH: * *p* < 0.05, ** *p* < 0.01, *** *p* < 0.001. Hashtags denote significance between non-ferrostatin pre-treated samples and ferrostatin pre-treated samples: ^#^ *p* < 0.05, ^##^ *p* < 0.01, ^###^ *p* < 0.001. (**E**) Representative immunoblot analysis of GPX4, NCOA4, KEAP1, and HMOX1 from WT- and IR-HG3 cells treated with SpiD3 (1, 2 μM), IBR (1 μM), FeCl_2_ (160 μM), or VEH for 4 h (*n* = 3 independent experiments/cell line). GAPDH served as the loading control. (**F**) Protein quantification of the immunoblot analysis of GPX4, NCOA4, KEAP1, and HMOX1. Data are represented as mean ± SEM. Asterisks denote significance vs. corresponding VEH: * *p* < 0.05, ** *p* < 0.01. Plus signs denote significance between WT-HG3 and IR-HG3 cells: ^+^ *p* < 0.05.

CRANAD2, a small-molecule probe that fluoresces when it is bound to aggregated proteins [52], was used to determine if SpiD3 would induce protein aggregation in WT- and IR-HG3 cells. SpiD3 dose-dependently increased CRANAD2 fluorescent intensity in both WT- and IR-HG3 cells (Figure 3A), implying an amplified burden of aggregated proteins in SpiD3-treated CLL cells. This result supports our earlier findings of increased unfolded protein levels in SpiD3-treated CLL cells [40]. Interestingly, ibrutinib did not alter CRANAD2 fluorescent intensity. Since genes related to oxidative stress and the NRF2/KEAP1 pathways were upregulated in SpiD3-treated WT- and IR-HG3 cells (Figure 2D–G), we sought to assess ROS production in SpiD3-treated CLL cells. In WT-HG3 cells, SpiD3 and ibrutinib both induced ROS production, while only 2 μM SpiD3 significantly induced ROS production in IR-HG3 cells (Figure 3B). Intriguingly, pre-treatment with the antioxidant NAC not only mitigated SpiD3- and ibrutinib-induced ROS production, but also attenuated SpiD3-mediated cytotoxicity (Figure 3B; Supplementary Figure S2A) in both WT- and IR-HG3 cells. IR-HG3 cells seemed to be more sensitive to SpiD3 treatment as there was a marked decrease in IR-HG3 cell viability compared to their WT counterparts (Supplementary Figure S2A).

To elucidate the etiology of SpiD3-induced ROS and the potential of SpiD3-related ROS to induce ferroptosis in WT- and IR-HG3 cells, the extent of lipid peroxidation was evaluated using the BODIPY-C11 581/591 flow cytometry probe [53]. Comparable to the ferroptosis inducer, FeCl_2_, SpiD3 treatment dose-dependently increased the percentage of peroxidized lipids in both WT- and IR-HG3 cells (Figure 3D). Interestingly, pre-treatment with ferrostatin did not restore viability in SpiD3-treated cells (Supplementary Figure S2B,C), suggesting that SpiD3 treatment could be inducing persistent stress, leading to greater cytotoxic outcomes in CLL cells. The expression of proteins vital to ferroptosis (GPX4, NCOA4) and ROS signaling (KEAP1 and HMOX1) was next evaluated in SpiD3-treated cells (Figure 3E). Comparable to FeCl_2_ treatment, SpiD3 treatment decreased GPX4 protein expression in WT-HG3 cells but only marginally decreased it in IR-HG3 cells. Interestingly, GPX4 protein expression was higher in IR-HG3 cells compared to WT-HG3 cells (Figure 3E,F), suggesting that these ibrutinib-resistant cells adapt to increased cellular stress. The expression of NCOA4, a marker for ferritinophagy and mediator of iron levels [18], was reduced in the WT- and IR-HG3 cells treated with either SpiD3 or FeCl_2_ (Figure 3E,F). SpiD3, but not FeCl_2_, decreased KEAP1 and increased HMOX1 protein levels in WT- and IR-HG3 cells (Figure 3E,F), indicating that SpiD3 can also induce oxidative stress, which may result in lipid peroxidation and the subsequent induction of ferroptosis in CLL.

### 3.3. SpiD3 Synergizes with Venetoclax and Displays Potent Cytotoxicity in Venetoclax-Resistant CLL Cells

**Figure 4.**
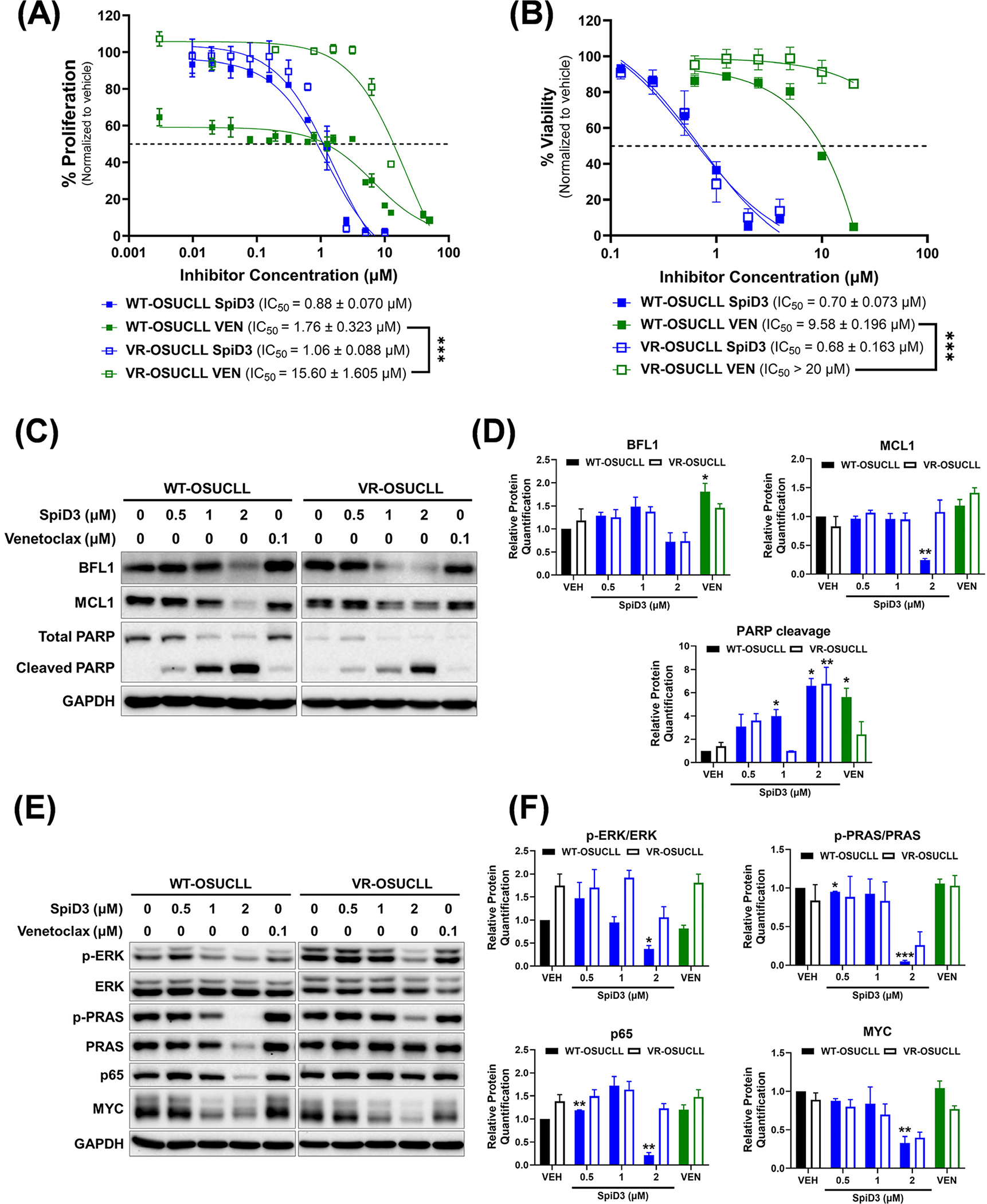
SpiD3 synergizes with venetoclax and elicits cytotoxic effects in venetoclax-resistant CLL cells. (**A**) Parental wild-type (WT) and venetoclax-resistant (VR) OSU-CLL cell proliferation was assessed by MTS assay following treatment with increasing concentrations of SpiD3 or venetoclax (VEN; *n* = 3 independent experiments/cell line) for 72 h. IC_50_ values (mean ± SEM) are noted for each cell line. (**B**) Percent viability of WT- and VR-OSUCLL cell lines was determined by Annexin V-FITC/PI viability assay following 24 h treatment with SpiD3 (*n* = 3 independent experiments/cell line). Asterisks indicate significance vs. corresponding vehicle. *** *p* < 0.001. (**C**) Representative immunoblot analysis of BFL1, MCL1, and PARP (total and cleaved) from WT- and VR-OSUCLL cells treated with SpiD3 (0.5, 1, 2 μM), venetoclax (0.1 μM), or equivalent DMSO vehicle (VEH) for 24 h (*n* = 3 independent experiments/cell line). GAPDH was used as the loading control. (**D**) Protein quantification of the immunoblot analysis of BFL1, MCL1, and cleaved PARP. Data are represented as mean ± SEM. Asterisks denote significance vs. corresponding VEH: * *p* < 0.05, ** *p* < 0.01. (**E**) Representative immunoblot analysis of p-ERK (Thr202/Tyr204), total ERK, p-PRAS (Thr246), total PRAS, p65, and MYC from WT- and VR-OSUCLL cells treated with SpiD3 (0.5, 1, 2 μM), VEN (0.1 μM), or VEH for 24 h (*n* = 3 independent experiments/cell line). GAPDH was used as the loading control (run on a separate blot). (**F**) Protein quantification of the immunoblot analysis of p-ERK (Thr202/Tyr204), p-PRAS (Thr246), p65, and MYC. Data are represented as mean ± SEM. Asterisks denote significance vs. corresponding VEH: * *p* < 0.05, ** *p* < 0.01, *** *p* < 0.001.

CLL cells rely on the overexpression of anti-apoptotic proteins, such as BCL2, for protection against spontaneous apoptosis [54]. Venetoclax, an FDA-approved BH3-mimetic molecule that binds with high affinity to BCL2, demonstrated impressive initial clinical activity; however, about 50% of high-risk CLL patients relapse after two years of venetoclax monotherapy [5]. Enhanced NF-κB activity was recently implicated in driving venetoclax resistance in CLL [15]. Accordingly, we investigated the cytotoxic effects of SpiD3 in VR-OSUCLL cells using cytotoxicity assays (MTS and Annexin-V/PI assays) and immunoblot analysis. The anti-proliferative and pro-apoptotic effects of venetoclax were abolished in VR-OSUCLL cells (Figure 4A,B). Strikingly, SpiD3 displayed robust anti-proliferative and pro-apoptotic effects in VR-OSUCLL cells with comparable IC_50_ to WT-OSUCLL cells (Figure 4A,B) [43]. On the protein level, SpiD3 decreased the anti-apoptotic proteins BFL1 and MCL1 and induced PARP cleavage in both WT- and VR-OSUCLL cell lines (Figure 4C,D). SpiD3 also decreased the phosphorylation of ERK and PRAS and decreased the protein expression of p65 and MYC in both WT- and VR-OSUCLL cell lines (Figure 4E,F). In contrast, venetoclax-induced effects were diminished in the resistant cells when compared to the wild-type cells (Figure 4C–F).

### 3.4. SpiD3 Alters Gene Expression of Key CLL Pathways in Venetoclax-Resistant CLL Cells

**Figure 5.**
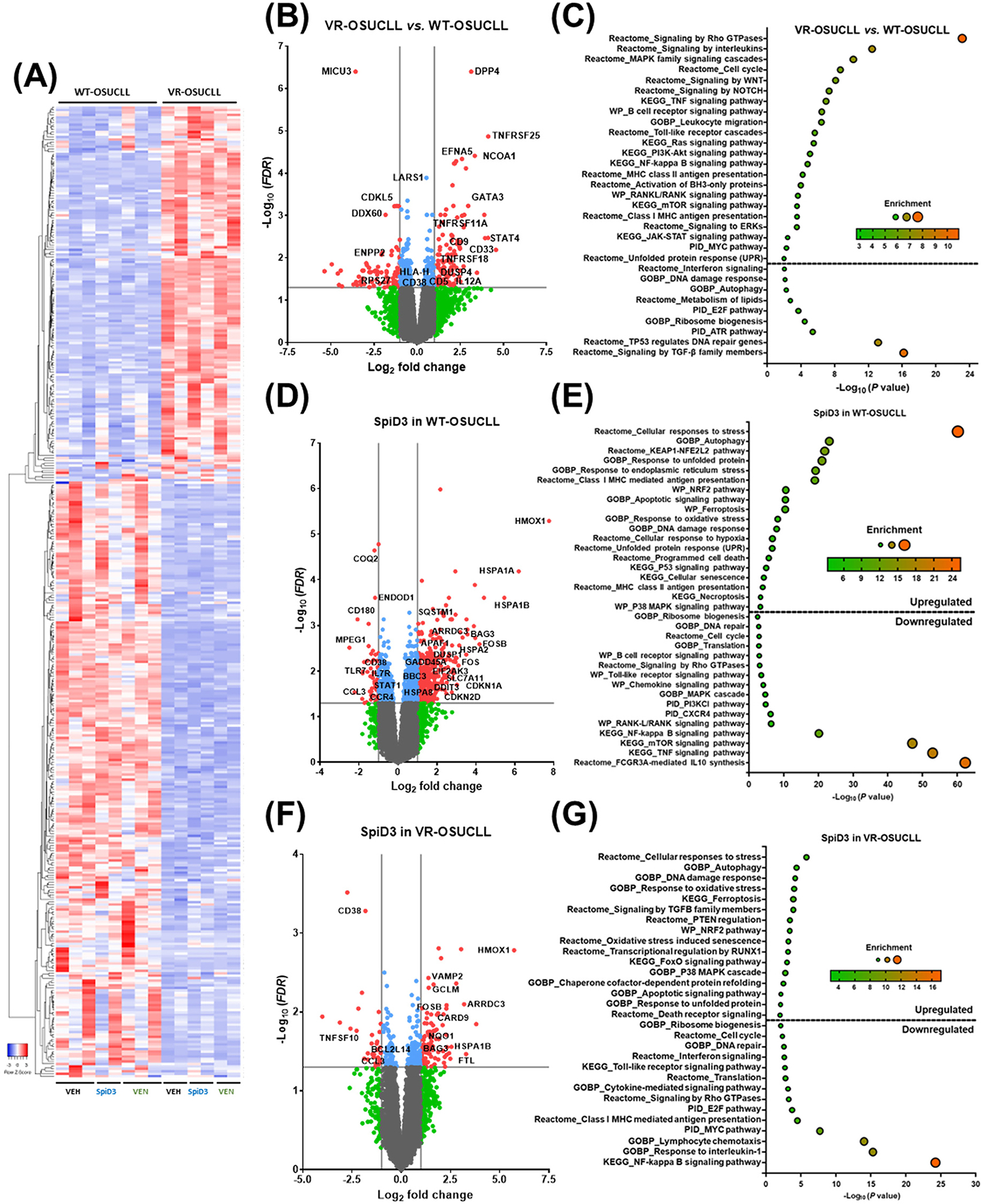
SpiD3 modifies transcriptional profiles in venetoclax-resistant CLL cells. (**A**–**G**) RNA-sequencing of parental wild-type (WT) OSU-CLL and venetoclax-resistant (VR) OSU-CLL cells treated with SpiD3 (2 μM), venetoclax (VEN; 1 μM), or equivalent DMSO vehicle (VEH; *n* = 2–3 independent experiments). (**A**) Hierarchical clustering of the top 500 differentially expressed genes (DEGs) in VEH-treated VR-OSUCLL cells compared to VEH-treated WT-OSUCLL (*FDR* < 0.05). Red indicates increased gene expression (0 < z-score < 2), and blueindicates decreased expression (−2 < z-score < 0). (**B**–**E**) The transcriptional profiles from vehicle- and 2 μM SpiD3-treated WT-OSUCLL samples from our previous study (GSE236239) [40] were incorporated into the volcano and gene set enrichment analysis (GSEA). (**B**,**D**,**F**) Volcano plots of VEH-treated VR-OSUCLL (**B**), SpiD3-treated WT-OSUCLL (**D**), or SpiD3-treated VR-OSUCLL cells (**F**) compared to VEH-treated OSU-CLL cells with select CLL-relevant genes labeled. Genes meeting both statistical significance (*FDR* < 0.05) and fold-change (|Log_2_ FC| > 1) parameters (red) were used for downstream analysis. Genes meeting only statistical significance (blue), only fold-change (green), or neither threshold (gray) are shown for comparison. (**C**,**E**,**G**) GSEA of statistically significant DEGs in VEH-treated VR-OSUCLL (**C**), SpiD3-treated WT-OSUCLL (**E**), and SpiD3-treated VR-OSUCLL cells (**G**) compared to VEH-treated OSU-CLL cells.

To probe deeper into the molecular characteristics of the venetoclax-resistant cells and the therapeutic effects of SpiD3 in VR-OSUCLL cells, we conducted transcriptomic analysis with WT- and VR-OSUCLL cells treated with either vehicle (DMSO), SpiD3, or venetoclax (Figure 5; Supplemental Figure S3), including transcriptional profiles from vehicle- and 2 μM SpiD3-treated WT-OSUCLL samples from our previous study (GSE236239) [40]. Transcriptional analysis revealed that over 500 genes (*FDR* < 0.05) were differentially expressed in VR-OSUCLL cells compared to WT-OSUCLL cells (Figure 5A,B). Various immune response-related genes were increased in VR-OSUCLL cells, such as *TNFRSF11A* (RANK), *CD33* (SIGLEC-3), *IL-12A*, *CD5*, *DUSP4*, and *TNFRSF18* (Figure 5A,B). Correspondingly, pathway enrichment analysis showed that “TNF signaling”, “TLR signaling”, “T-cell activation”, “NF-κB signaling”, “RANK-L/RANK signaling”, and “MHC class I and class II antigen processing and presentation” were upregulated in VR-OSUCLL cells (Figure 5C), highlighting potential vulnerabilities in venetoclax-resistant CLL cells. As seen with IR-HG3 cells, pathway enrichment analysis revealed that genes in the “signaling by RHO GTPase”, “MAPK signaling”, “WNT/β-catenin signaling”, and “NOTCH signaling” pathways were also upregulated in VR-OSUCLL cells. Interestingly, the “BH3-only proteins” pathway was upregulated in VR-OSUCLL, indicating a compensatory mechanism to override BCL2 inhibition (Figure 5C). Genes involved in the “RAS signaling”, “PI3K/AKT signaling”, mTOR signaling”, “MYC activation”, and “UPR signaling” pathways were also upregulated in venetoclax-resistant cells, further supporting the upregulation of alternative signaling pathways to drive venetoclax resistance in CLL (Figure 5B,C). Lastly, the VR-OSUCLL cells seem to be less dependent on nutrient recycling, as genes involved in “autophagy” and “metabolism of lipids” were downregulated (Figure 5B,C).

Venetoclax upregulated genes involved in the “apoptosis” pathway and downregulated genes involved in the “translation” and “MHC class I and II antigen presentation” pathways in WT-OSUCLL cells but not in VR-OSUCLL cells, which suggests reduced sensitivity to venetoclax-induced gene expression changes in resistant cells (Supplementary Figure S3). In both the WT- and VR-OSUCLL cells, SpiD3 increased the expression of genes involved in the “cellular response to stress”, “autophagy”, “DNA damage”, and “apoptosis” pathways (Figure 5D–G). Additionally, the “cell cycle”, “TLR signaling”, and “DNA repair” pathways were decreased in SpiD3-treated WT- and VR-OSUCLL cell lines (Figure 5D–G). Genes associated with the “ferroptosis”, “NRF2 signaling”, and “cellular response to oxidative stress” pathways (*HMOX1*, *GCLM*, and *NQO1*) were also upregulated in SpiD3-treated WT- and VR-OSUCLL cells, indicating that SpiD3 may induce ROS signaling, impairing the survival of CLL cells. Interestingly, pathways that were upregulated in VR-OSUCLL cells, such as “mTOR/translation signaling”, “cell cycle”, “NF-κB signaling”, and “signaling by Rho GTPase”, were downregulated with SpiD3 in both WT- and VR-OSUCLL cells (Figure 5D–G), suggesting that SpiD3 can target alternative survival mechanisms/pathways commonly associated with drug resistance mechanisms. These results highlight the ability of SpiD3 to modulate various pro-survival and anti-apoptotic pathways in venetoclax-resistant CLL cells.

### 3.5. SpiD3 Modulates Critical Pathways in Venetoclax-Resistant CLL Cells

**Figure 6.**
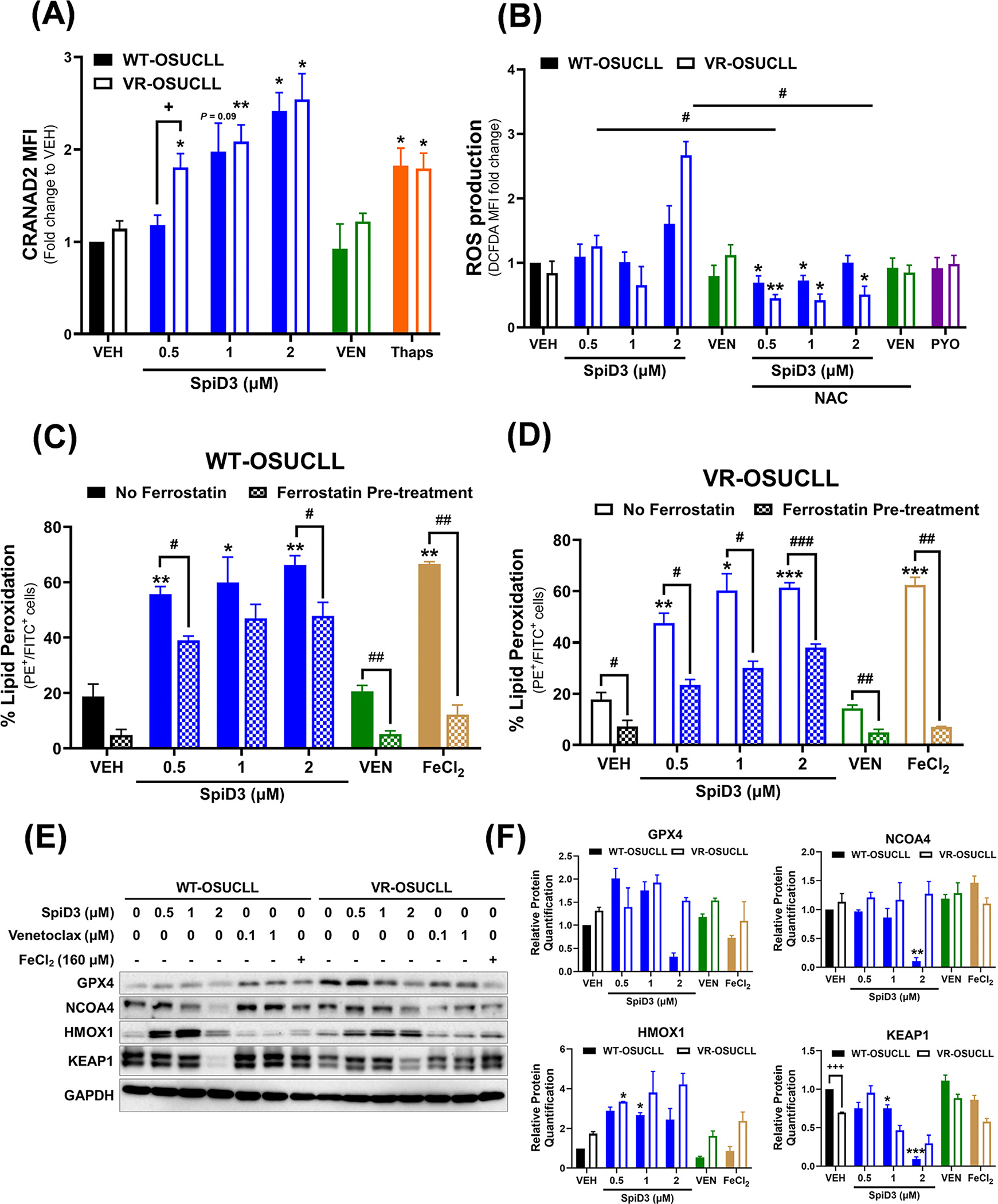
SpiD3 subverts oncogenic pathways in venetoclax-resistant CLL cells. (**A**) Parental wild-type (WT) and venetoclax-resistant (VR) OSU-CLL cells were treated for 24 h with SpiD3 (0.5, 1, 2 μM), thapsigargin (Thaps; 2 μM), venetoclax (VEN; 1 μM), or equivalent DMSO vehicle (VEH) and then incubated with CRANAD2 dye to probe for aggregate proteins. Data are shown as fold-change in CRANAD2 median fluorescent intensity (MFI) compared to VEH-treated WT-OSUCLL cells (*n* = 3 independent experiments/cell line). Data are represented as mean ± SEM. Asterisks denote significance vs. corresponding VEH: * *p* < 0.05, ** *p* < 0.01. The plus sign denotes significance between WT-OSUCLL and VR-OSUCLL cells: ^+^ *p* < 0.05. (**B**) WT-OSUCLL and VR-OSUCLL cells were pre-treated with 5 mM N-acetylcysteine (NAC, 1 h), followed by SpiD3 (0.5, 1, 2 μM), VEN (1 μM), or VEH (24 h). Pyocyanin (PYO, 1 mM) served as a control ROS inducer (*n* = 3 independent experiments/cell line). ROS production is represented as fold-change in DCFDA MFI to VEH-treated WT-OSUCLL cells. Data are represented as mean ± SEM. Asterisks denote significance vs. corresponding VEH: * *p* < 0.05, ** *p* < 0.01. Hashtags denote significance between non-NAC pre-treated samples and NAC pre-treated samples: ^#^ *p* < 0.05. (**C**,**D**) WT-OSUCLL (**C**) and VR-OSUCLL (**D**) cells were pre-treated with 10 μM ferrostatin (1 h; inhibitor of lipid peroxidation) followed by SpiD3 (0.5, 1, 2 μM), VEN (1 μM), or VEH (48 h). Iron (II) chloride tetrahydrate (FeCl_2_; 160 μM) served as a control lipid peroxidation inducer (*n* = 3 independent experiments/cell line). Cells were analyzed by flow cytometry for changes in peroxidized lipid generation, observed by a change in fluorescence from PE to FITC. Data are represented as mean ± SEM. Asterisks denote significance vs. corresponding VEH: * *p* < 0.05, ** *p* < 0.01, *** *p* < 0.001. Hashtags denote significance between non-ferrostatin pre-treated samples and ferrostatin pre-treated samples: ^#^ *p* < 0.05, ^##^ *p* < 0.01, ^###^ *p* < 0.001. (**E**) Representative immunoblot analysis of GPX4, NCOA4, KEAP1, and HMOX1 from WT- and VR-OSUCLL cells treated with SpiD3 (1, 2 μM), VEN (0.1, 1 μM), FeCl_2_ (160 μM), or VEH for 24 h (n = 3 independent experiments/cell line). GAPDH served as the loading control. (**F**) Protein quantification of the immunoblot analysis of GPX4, NCOA4, KEAP1, and HMOX1. Data are represented as mean ± SEM. Asterisks denote significance vs. corresponding VEH: * *p* < 0.05, ** *p* < 0.01, *** *p* < 0.001. Plus signs denote significance between WT-OSUCLL and VR-OSUCLL cells: ^+++^ *p* < 0.001.

Functional flow cytometry assays were used to validate the transcriptional profiling (RNA-sequencing) findings through the measurement of protein aggregation and ROS production. SpiD3 dose-dependently increased CRANAD2 fluorescent intensity in both WT- and VR-OSUCLL cells (Figure 6A), implying an amplified burden of aggregated proteins in SpiD3-treated CLL cells comparable to thapsigargin, a control UPR inducer. In VR-OSUCLL cells, ROS production was slightly increased with 2 μM SpiD3 treatment (Figure 6B). Interestingly, pre-treatment with the antioxidant, NAC, mitigated SpiD3-induced ROS production while attenuating SpiD3-mediated cytotoxicity in WT- and VR-OSUCLL cells (Figure 6B; Supplementary Figure S4A). SpiD3 also induced unreconcilable lipid peroxidation dose-dependently following 48 h of treatment in both WT- and VR-OSUCLL cells (Figure 6C,D). Similar to ibrutinib-resistant cells, pre-treatment with ferrostatin did not restore viability in SpiD3-treated cells (Supplementary Figure S4B,C), emphasizing that SpiD3 can induce ferroptosis through various pathways. GPX4 and NCOA4 protein expression was reduced within 24 h SpiD3 treatment, indicating that SpiD3 can induce ferroptosis (Figure 6E,F). HMOX1 protein expression increased with SpiD3 treatment, while KEAP1 protein expression dose-dependently decreased with SpiD3 treatment in both WT- and VR-OSUCLL cells (Figure 6E,F), indicating the induction of oxidative stress. In conclusion, our results demonstrate that SpiD3 is a potential therapeutic lead for aggressive, relapsed/refractory CLL as it can circumvent previously reported drug-resistant mechanisms [5,7,9].

## 4. Discussion

In this study, we revealed distinct molecular characteristics of ibrutinib and venetoclax-resistant CLL cells. We also expanded our understanding on the mechanism of action of SpiD3 in CLL. Remarkably, SpiD3 retained its antitumor effects in both ibrutinib- and venetoclax-resistant cells and displayed a strong ferroptotic signature, accompanied by UPR induction and ROS signaling culminating to CLL cell death.

Previously, we used an alkyne-tagged analog 19 to pull down potential proteins that could interact with SpiD3 [40]. KEAP1, SLC3A2, and GPX4 were identified in the mass spectrometry analysis (PXD043688) [40], indicating that SpiD3 could directly bind to these proteins. KEAP1 is involved in resolving oxidative stress, and reduced KEAP1 expression leads to increased oxidative stress. Consistent with our previous results [40], SpiD3 induced ROS production and increased HMOX1 protein expression in WT- and inhibitor-resistant CLL cells. Due to its covalent interactions with SECs [40], SpiD3 could potentiate ferroptosis by binding to free cyst(e)ine or interfering with its import by disrupting the interaction between SLC3A2 and SLC7A11 heterodimers, effectively sequestering cyst(e)ine from GSH production and depleting the GSH antioxidant pool, which results in unattenuated peroxidized lipid accumulation. The lower molecular weight band of SLC3A2 observed in the immunoblot analysis of SpiD3-treated cells (Supplementary Figure S5) indicates a monomer form of SLC3A2 instead of homodimer/heterodimer form [55], highlighting the potential of SpiD3 to interfere with cyst(e)ine import.

In the inhibitor-resistant cell lines, SpiD3 increased genes involved in the UPR pathway and induced protein aggregation. This indicates that SpiD3 retains its ability to crosslink proteins in ibrutinib- and venetoclax-resistant CLL cells, warranting further studies to validate the UPR signature witnessed in SpiD3-treated inhibitor-resistant CLL cells. Additionally, genes involved in MHC class I and II presentation and antigen processing were upregulated in both SpiD3-treated resistant cell lines. Previous studies have shown that covalent drug–protein binding can generate hapten-like peptides that stimulate cytotoxic T-cells when presented by MHC class I [56–58]. Further studies are warranted to verify SpiD3’s potential to induce an immune response in drug-resistant CLL.

As mentioned previously, a common resistance mechanism to venetoclax treatment is the upregulation of anti-apoptosis factors and downregulation of pro-apoptosis factors [59]. Additionally, anti-apoptotic MCL1 and BCL-xL protein expression can be upregulated by NF-κB activation [15]. In venetoclax-resistant CLL cells, genes involved in extrinsic and intrinsic apoptotic pathways were upregulated. In SpiD3-treated venetoclax-resistant CLL cells, the expression of *TNFSF10* (TRAIL) and *BCL2L14* anti-apoptotic genes was reduced, indicating that SpiD3 can modulate venetoclax-resistant apoptosis mechanisms. In ibrutinib-resistant CLL cells, SpiD3 treatment decreased MCL1 protein expression, indicating reduced sequestration of pro-apoptotic factors.

Phase 2 (CAPTIVATE; NCT02910583) and phase 3 (GLOW; NCT03462719) clinical trials of ibrutinib and venetoclax combination for CLL have shown promising clinical results, highlighting the therapeutic potential of concurrently inhibiting BCR → NF-κB signaling and apoptotic signaling in CLL [60,61]. NOTCH, WNT, and Rho GTPase signaling were upregulated in both non-treated ibrutinib- and venetoclax-resistant cells, suggesting that there may be overlap in resistance mechanisms to ibrutinib and venetoclax treatment in CLL cells. Notably, SpiD3 synergized with venetoclax at all the concentrations tested, and we previously reported similar findings with ibrutinib [40], suggesting that SpiD3 mediated the induction of ferroptosis/UPR activation as a promising approach to treat drug-resistant CLL. Future studies in double-refractory disease models that evaluate novel agents, such as SpiD3, in comparison to the combination of ibrutinib and venetoclax, may provide alternative therapeutic strategies. Altogether, these data support the development of SpiD3 as a novel therapeutic for relapsed/refractory CLL patients.

## 5. Patents

A.N. and S.R. hold a patent for SpiD3 as novel dimers of covalent NF-κB inhibitors (US 2019/0322680 A1, Natarajan et al., 2019) [62].

## Supplementary Material

Supplementary Material

## Data Availability

RNA-sequencing data are deposited at GSE267095. WT-OSUCLL samples from GSE236239 (GSM7520211, GSM7520213, GSM7520214, GSM7520216, GSM7520217, GSM7520219) [40] were analyzed with the vehicle- and SpiD3-treated WT-OSUCLL cells from GSE267095.
