# Supplementary material for: The Novel Anti-Cancer Agent, SpiD3, Is Cytotoxic in CLL Cells Resistant to Ibrutinib or Venetoclax
Source: Hemato. Author manuscript; Available in PMC 2024 Oct 24. (PMC11500768; doi:10.3390/hemato5030024)
Supplement: Supplementary Material [file NIHMS2026135-supplement-Supplementary_Material.pdf]

Supplemental Tables and Figures

Supplemental Table S1: Molecular characteristics of the CLL cell lines.

CLL cell line	Key characteristics (*IGHV* mutational status & karyotype)
HG-3[1]	Mutated IGHV; Non-complex karyotype; t12, t19
OSU-CLL[2]	Unmutated IGHV; Non-complex karyotype; del 13q

IGHV: immunoglobulin heavy-chain variable region gene.
[1] The HG-3 cell line originated from a CLL patient with un-mutated IGHV and demonstrates non-complex karyotype with biallelic 13q14 deletions. [1]
[2] The OSU-CLL cell line originated from a CLL patient with mutated IGHV and non-complex karyotype that is defined as having less than three chromosomal aberrations. [2]

Supplemental Table S2: Primary antibodies for immunoblotting.

Primary Antibodies	Catalog number
**Cell Signaling Technology**	
BFL1	14093
p-BTK (Tyr223)	87457
BTK	8547
p-ERK1/2 (Thr202/Tyr204)	4377
ERK1/2	4695
GAPDH	5174
GPX4	52455
HMOX1	43966
KEAP1	8047
MCL1	5453
c-MYC	5605
NCOA4	66849
PARP	9542
p-PRAS (Thr246)	13175
PRAS	2691
SLC3A2	47213
**Santa Cruz Biotechnology**	
p65	sc-8008

Supplemental Figure S1: Ibrutinib modifies transcriptional profiles in wild-type and ibrutinib-resistant CLL cells.

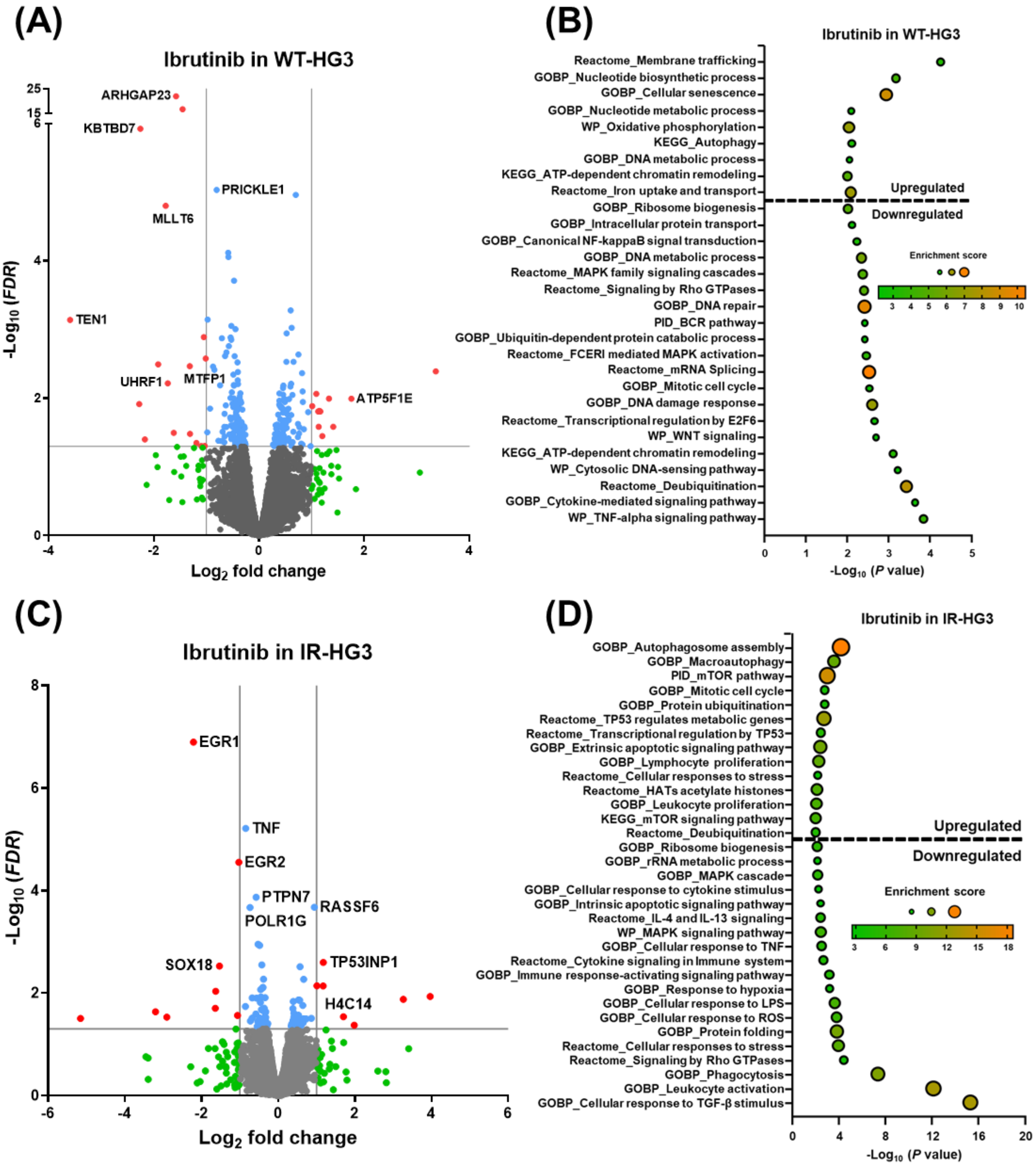

Supplemental Figure S1: Ibrutinib modifies transcriptional profiles in wild-type and ibrutinib-resistant CLL cells. (**A-D**): RNA-sequencing of parental wild-type (WT) HG-3 and ibrutinib-resistant (IR) HG-3 cells treated with ibrutinib (1 μM) or equivalent DMSO vehicle (VEH; *n* = 3 independent experiments). (**A**) Volcano plot of ibrutinib-treated WT-HG3 cells compared to VEH-treated WT-HG3 cells with select CLL-relevant genes labeled. Genes meeting both the statistical significance (*FDR* < 0.05) and fold-change ($|\text{Log}_2 \text{FC}| > 1$) parameters (red) were used for downstream analysis. Genes meeting only statistical significance

(blue), only fold-change (green), or neither threshold (grey) are shown for comparison. (**B**) Gene set enrichment analysis (GSEA) of the statistically significant differentially expressed genes (DEGs) in ibrutinib-treated WT-HG3 cells compared to VEH-treated WT-HG3 cells. (**C**) Volcano plot of ibrutinib-treated IR-HG3 cells compared to VEH-treated IR-HG3 cells with select CLL-relevant genes labeled. Genes meeting both the statistical significance (*FDR* < 0.05) and fold-change ($|Log_2 FC| > 1$) parameters (red) were used for downstream analysis (red). Genes meeting only statistical significance (blue), only fold-change (green), or neither threshold (grey) are shown for comparison. (**D**) GSEA of the statistically significant DEGs in ibrutinib-treated IR-HG3 cells compared to VEH-treated IR-HG3 cells.

Supplemental Figure S2: SpiD3 decreases viability while modulating ferroptosis in ibrutinib-resistant CLL cells.

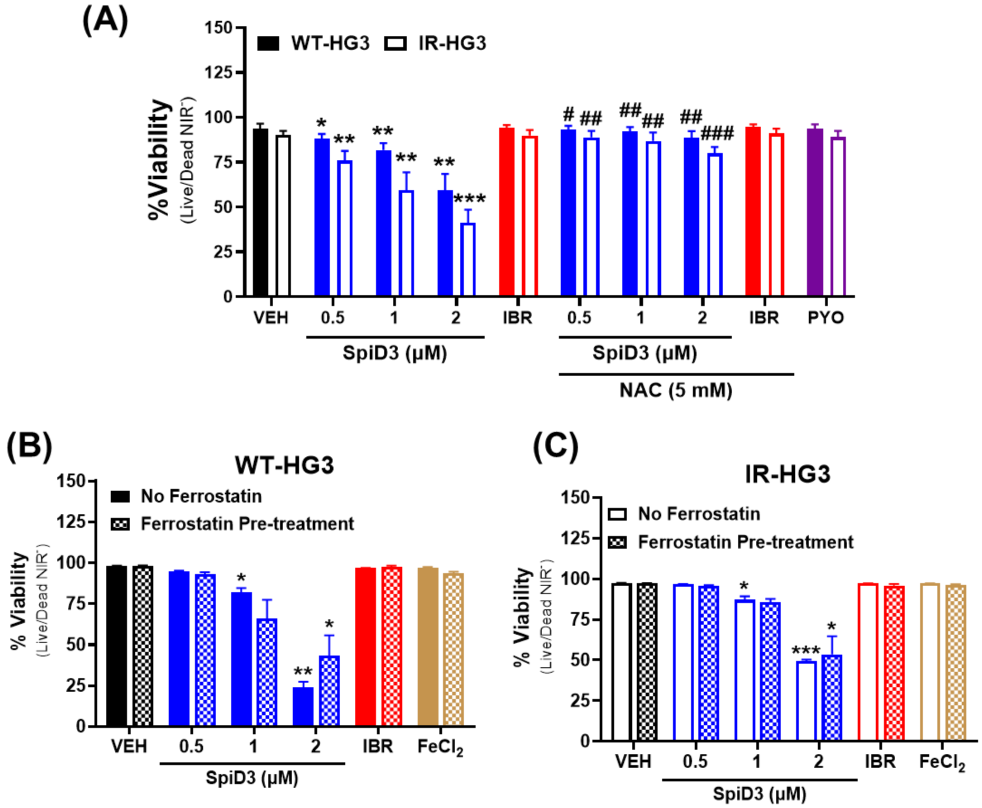

Supplemental Figure S2: SpiD3 decreases viability while modulating ferroptosis in ibrutinib-resistant CLL cells. (**A**) Wild-type (WT) and ibrutinib (IR) HG-3 cells were pre-treated with 5 mM N-acetylcysteine (NAC, 1 h) followed by SpiD3 (0.5, 1, 2 μM), ibrutinib (IBR; 1 μM), or equivalent DMSO vehicle (VEH) for 24 h. Pyocyanin (PYO, 1 mM) served as a control ROS inducer (n = 3 independent experiments/cell line). Percent viability per condition is shown. Data are represented as mean ± SEM. Asterisks denote significance vs. corresponding VEH: * $p < 0.05$, ** $p < 0.01$, *** $p < 0.001$. Hashtags denote significance between non-NAC pre-treated samples and NAC pre-treated samples: $^{\#}p < 0.05$, $^{\#\#}p < 0.01$, $^{\#\#\#}p < 0.001$. (**B,C**): WT-HG3 (**B**) and IR-HG3 (**C**) cells were pre-treated with ferrostatin (10 μM) for 1 h followed by SpiD3 (0.5, 1, 2 μM), IBR (1 μM), or VEH for 48 h. $FeCl_2$ (160 μM) served as a control ferroptosis inducer. Percent viability per condition is shown. Data are represented as mean ± SEM. Asterisks denote significance vs. corresponding VEH: * $p < 0.05$, ** $p < 0.01$, *** $p < 0.001$.

Supplemental Figure S3: Venetoclax modifies transcriptional profiles in wild-type and venetoclax-resistant cells.

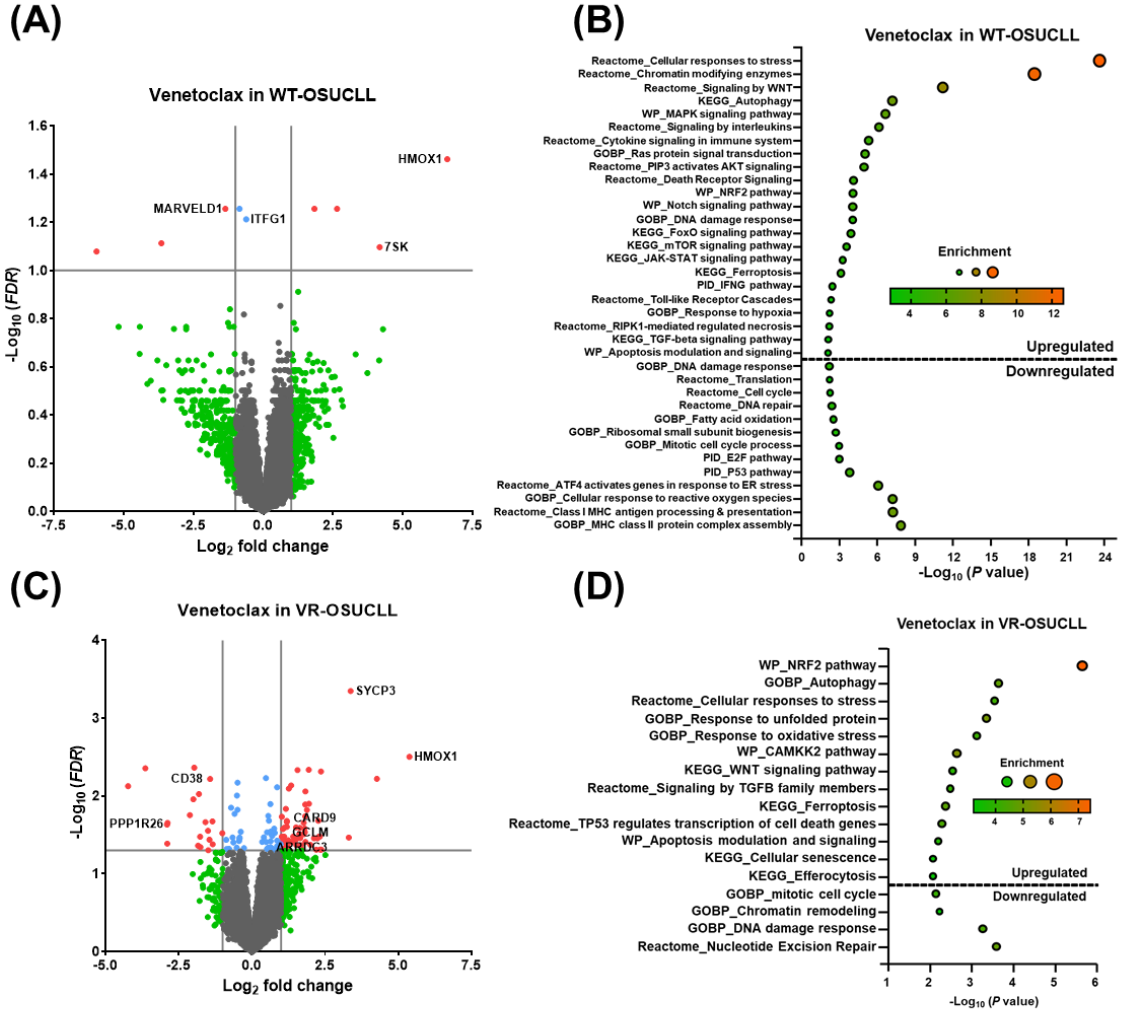

Supplemental Figure S3: Venetoclax modifies transcriptional profiles in wild-type and venetoclax-resistant cells. (**A-D**): RNA-sequencing of parental wild-type (WT) OSU-CLL and venetoclax-resistant (VR) OSU-CLL cells treated with venetoclax (1 μM) or equivalent DMSO vehicle (VEH; *n* = 3 independent experiments). (**A,B**): The transcriptional profiles from vehicle-treated WT-OSUCLL samples from the previous study (GSE236239) [3] were analyzed and incorporated into the volcano and gene set enrichment analysis (GSEA). (**A**) Volcano plot of venetoclax-treated WT-OSUCLL cells compared to VEH-treated WT-OSUCLL cells with select CLL-relevant genes labeled. Genes meeting both the statistical significance (*FDR* < 0.05) and fold-change (|Log_2 FC| > 1) parameters (red) were used for downstream analysis. Genes meeting only statistical significance (blue), only fold-change (green), or neither threshold (grey) are shown for comparison. (**B**) GSEA of the statistically significant differentially expressed genes (DEGs) in venetoclax-treated WT-OSUCLL cells compared to VEH-treated WT-OSUCLL cells. (**C**) Volcano plot of venetoclax-treated VR-OSUCLL cells compared to VEH-treated VR-OSUCLL cells with select CLL-relevant genes labeled. Genes meeting both the statistical significance (*FDR* < 0.05) and fold-change (|Log_2 FC| > 1) parameters (red) were used for downstream analysis. Genes meeting only statistical significance (blue), only fold-change (green), or neither threshold (grey) are shown for comparison. (**D**) GSEA of the statistically significant DEGs in venetoclax-treated VR-OSUCLL cells compared to VEH-treated VR-OSUCLL cells.

Supplemental Figure S4: SpiD3 decreases viability while modulating ferroptosis in venetoclax-resistant CLL cells.

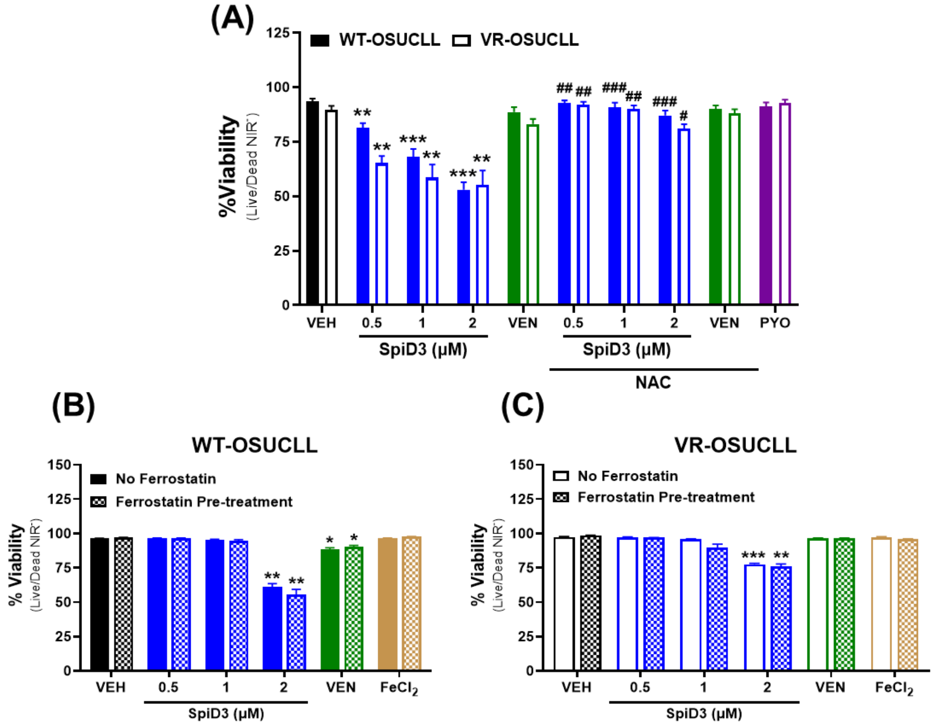

Supplemental Figure S4: SpiD3 decreases viability while modulating ferroptosis in venetoclax-resistant CLL cells. (**A**) Wild-type **(**WT) and venetoclax-resistant (VR) OSU-CLL cells were pre-treated with 5 mM N-acetylcysteine (NAC, 1 h) followed by SpiD3 (0.5, 1, 2 μM), venetoclax (VEN; 1 μM), or equivalent DMSO vehicle (VEH) for 24 h. Pyocyanin (PYO, 1 mM) served as a control ROS inducer (*n* = 3 independent experiments/cell line). Percent viability per condition is shown. Data are represented as mean ± SEM. Asterisks denote significance vs. corresponding VEH: ** $p < 0.01$, *** $p < 0.001$. Hashtags denote significance between non-NAC pre-treated samples and NAC pre-treated samples: $^{\#}$ $p < 0.05$, $^{\#\#}$ $p < 0.01$, $^{\#\#\#}$ $p < 0.001$. (**B**,**C**): WT-OSUCLL (**B**) and VR-OSUCLL (**C**) cells were pre-treated with ferrostatin (10 μM) for 1 h followed by SpiD3 (0.5, 1, 2 μM), VEN (1 μM), or VEH for 48 h. $FeCl_2$ (160 μM) served as a control ferroptosis inducer. Percent viability per condition is shown. Data are represented as mean ± SEM. Asterisks denote significance vs. corresponding VEH: * $p < 0.05$, ** $p < 0.01$, *** $p < 0.001$.

Supplemental Figure S5: Protein quantification of SLC3A2.

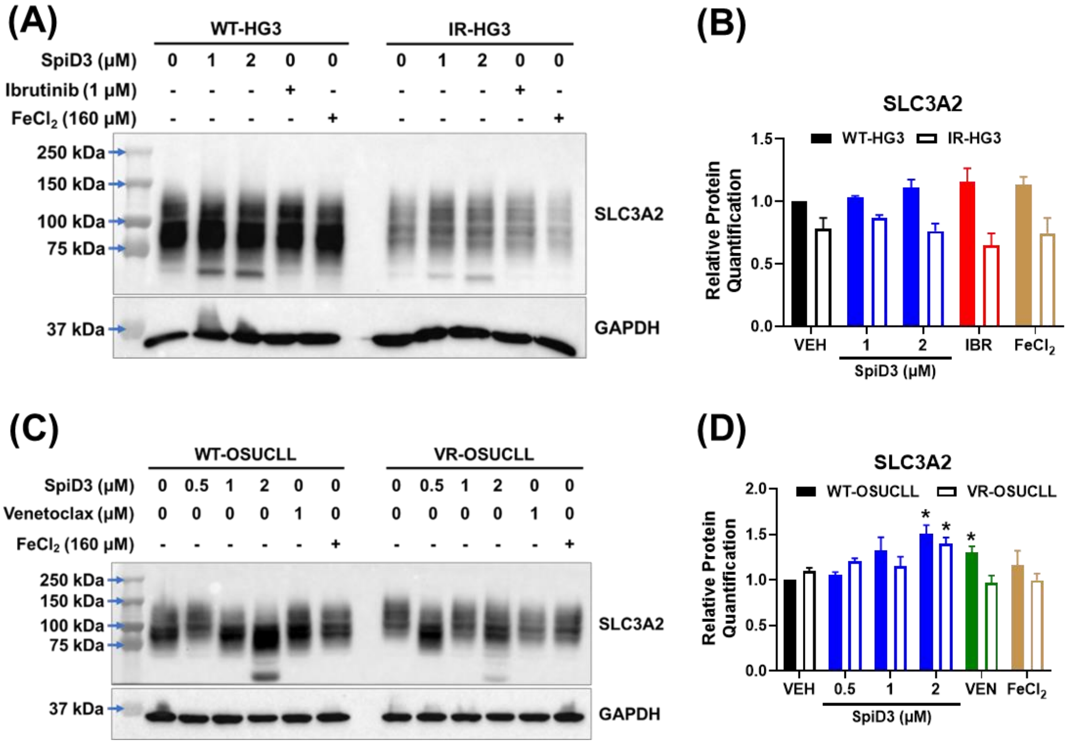

Supplemental Figure S5: Protein quantification of SLC3A2. (**A**) Representative immunoblot analysis of SLC3A2 in WT-HG3 and IR-HG3 cells treated with SpiD3 (1, 2 μM), ibrutinib (1 μM), or $FeCl_2$ (160 μM) for 4 h (*n* = 3 independent experiments). GAPDH served as the loading control. (**B**) Protein quantification of the immunoblot analysis of SLC3A2. Data are represented as mean ± SEM. (**C**) Representative immunoblot analysis of SLC3A2 in WT-OSUCLL and VR-OSUCLL cells treated with SpiD3 (0.5, 1, 2 μM), VEN (1 μM), or $FeCl_2$ (160 μM) for 24 h (*n* = 3 independent experiments/cell line). GAPDH served as the loading control. (**D**) Protein quantification of the immunoblot analysis of SLC3A2. Data are represented as mean ± SEM. Asterisks denote significance vs. corresponding VEH: * $p < 0.05$.

References

1. Rosen, A.; Bergh, A.C.; Gogok, P.; Evaldsson, C.; Myhrinder, A.L.; Hellqvist, E.; Rasul, A.; Bjorkholm, M.; Jansson, M.; Mansouri, L.; et al. Lymphoblastoid cell line with B1 cell characteristics established from a chronic lymphocytic leukemia clone by in vitro EBV infection. *Oncoimmunology* **2012**, *1*, 18-27, doi:10.4161/onci.1.1.18400.
2. Hertlein, E.; Beckwith, K.A.; Lozanski, G.; Chen, T.L.; Towns, W.H.; Johnson, A.J.; Lehman, A.; Ruppert, A.S.; Bolon, B.; Andritsos, L.; et al. Characterization of a new chronic lymphocytic leukemia cell line for mechanistic in vitro and in vivo studies relevant to disease. *PLoS One* **2013**, *8*, e76607, doi:10.1371/journal.pone.0076607.
3. Eiken, A.P.; Smith, A.L.; Skupa, S.A.; Schmitz, E.; Rana, S.; Singh, S.; Kumar, S.; Mallareddy, J.R.; de Cubas, A.A.; Krishna, A.; et al. Novel spirocyclic dimer, SpiD3, targets chronic lymphocytic leukemia survival pathways with potent preclinical effects. *Cancer Res Commun* **2024**, *4*, 1328-1343, doi:10.1158/2767-9764.CRC-24-0071.
